# Risk factors for high-risk and multi-type Human Papillomavirus infections among women in Ho Chi Minh City, Vietnam: a cross-sectional study

**DOI:** 10.1186/s12905-015-0172-7

**Published:** 2015-02-21

**Authors:** Ly Thi-Hai Tran, Loi Thi Tran, Thanh Cong Bui, Dung Thi-Kieu Le, Alan G Nyitray, Christine M Markham, Michael D Swartz, Chau Bao Vu-Tran, Lu-Yu Hwang

**Affiliations:** Department of Epidemiology, School of Public Health, The University of Texas Health Science Center at Houston, Houston, TX USA; Department of Obstetrics and Gynecology, Faculty of Medicine, Vietnam National University in Ho Chi Minh City, Ho Chi Minh City, Vietnam; Department of Behavioral Science, The University of Texas MD Anderson Cancer Center, Houston, TX USA; Department of Obstetrics and Gynecology, University of Medicine and Pharmacy in Ho Chi Minh City, Ho Chi Minh City, Vietnam; Department of Health Promotion and Behavioral Sciences, School of Public Health, The University of Texas Health Science Center at Houston, Houston, TX USA; Department of Biostatistics, School of Public Health, The University of Texas Health Science Center at Houston, Houston, TX USA

**Keywords:** High-risk HPV infection, Multiple HPV infection, Risk factors, Vietnam

## Abstract

**Background:**

Concurrent infection with multiple types of Human Papillomavirus (HPV) is associated with an increased risk of cervical cancer; yet, little is known about risk factors for concurrent HPV infection in Vietnam. This study investigated the prevalence of and risk factors for high-risk-type HPV and multi-type HPV infections among women in Ho Chi Minh City, Vietnam.

**Methods:**

Data were collected from a population-based survey of 1,550 women (mean age = 42.4; SD = 9.5), using a multi-stage sampling process. Socio-demographic and behavioral variables were obtained by self-report. HPV genotypes in cervical specimens were identified using PCR protocols.

**Results:**

The prevalence of any high-risk HPV infection was 9.0%, and of multi-type HPV infection was 1.9%. In the HPV+ subsample, the percentage of high-risk HPV was 84% and of multi-type HPV was 20%. All multi-type HPV infections were high-risk-type. Lifetime smoking and older age of first sex were significantly associated with any high-risk and multi-type HPV infections. Regular condom use was inversely associated with high-risk and multi-type HPV infection.

**Conclusions:**

Risk factors for high-risk and multi-type HPV infections were similar. Further research and intervention are needed to reduce HPV infections in order to prevent HPV-related cancers.

## Background

Human Papillomavirus (HPV) infection, particularly persistent infection with high-risk types, is a major preventable causal agent of cervical cancer [1,2]. More than 100 types of HPVs are known, of which 18 types are classified as high risk (i.e., oncogenic) [3]. Persistent infection with high-risk HPV types has been shown to increase residual or recurrent cervical disease in women after surgical treatment for high-grade cervical lesions [4]. In the International Agency for Research on Cancer (IARC) data for Vietnam in 2003, the prevalence of any HPV genotype was 2.0% in Hanoi and 10.9% in Ho Chi Minh City (HCMC) [5]. Several studies have examined risk factors for genital HPV infection, particularly with high-risk types. These risk factors include lifetime number of sexual partners, having a new partner, and partner’s sexual history [6]. Other less consistent determinants are age of sexual debut, smoking, parity, and oral contraceptive use [2,6].

Concurrent infection with multiple HPV types is common. In a pooled analysis in 15 areas worldwide, the prevalence of multi-type HPV infection ranged from 0.3–11.8% [7]. Among HPV-positive women, this prevalence averaged about 25% and ranged from 18.5–46% [7-9]. A number of studies have observed associations between multi-type HPV infection and certain health consequences, such as abnormal Pap results [10,11], high-grade squamous intraepithelial lesions [12,13], and a nearly 5-fold higher proportion of failure in radiotherapy treatment in cervical cancer patients [14]. The increase in risk for cervical intraepithelial neoplasia and high-grade squamous intraepithelial lesions associated with multi-type HPV infection may be comparable to the sum of risk caused by individual types and there is no evidence for synergistic interactions [8]. Risk factors for multi-type infection have also been examined in a few studies. The variance in multi-type HPV prevalence in all surveyed women and in HPV+ women across different regions of the world suggested that there might be some different risk factors for multi-type HPV infection compared to any-type HPV infection. Higher numbers of sexual partners were observed for those who were infected with high-risk HPV and multi-type HPV compared to those who were infected with low-risk type and single-type, respectively; yet, these were not statistically significant [15]. An effect of condom use on single-type and multi-type HPV infections was also not statistically significant. Thus, it is necessary to continue investigating risk factors for multi-type HPV infections.

Although a few studies have reported risk factors associated with general HPV infection in HCMC, Vietnam [5], little is known about risk factors of infection with high-risk and multiple HPV types. In our previous report, the unweighted HPV prevalence in women in HCMC was 10.8%, of which the most common types were 16, 18, and 58 [16]. The current analysis was conducted to investigate the weighted prevalence of and risk factors for high-risk-type and multi-type HPV infection. Because this survey employed probability sampling proportional to size sampling, the weighted procedure may produce more accurate results than unweighted analyses. Results of this analysis will contribute important evidence regarding knowledge of the determinants of HPV infection, particularly with multiple types, and may inform future prevention strategies to reduce HPV infection and subsequent cancer risks.

## Methods

### Study design, population, and sampling

This study employed data from a population-based household survey of 1,550 women in HCMC, which was conducted from April 2008 to January 2009 to examine the predictive values of using an HPV test and/or a Pap smear in cervical cancer screening and diagnosis. Participants were selected through a two-stage sampling process. First, 10 districts (out of 24) and two communes (out of an average of 16) in each district in HCMC were randomly selected. Then, in each district, individuals were sampled where the probability of being selected was proportional to the size of the ward, based on the commune police registration lists. Women were eligible if they ever had sexual intercourse, were 18–69 years old, were registered residents of HCMC, and did not suffer from mental impairment. Exclusion criteria included women who were pregnant, had undergone a hysterectomy or a treatment for potential cervical cancer; had abnormal vaginal bleeding, or had any obstetrical/gynecological emergency. Selected women were fully informed about the purpose of the parent study, and the benefits/risks of participation. Women who agreed to participate provided written informed consents, and were invited to come to the commune health stations for a brief 15-minute interview and a gynecological examination, including collecting specimens for HPV tests and Pap tests. Interviews to obtain participants’ demographic and behavioral characteristics and medical history were conducted in Vietnamese in a private place (e.g., an empty room) at the commune health stations. Cervical specimens were then collected by trained gynecologists. Each participant received compensation (equivalent to $2.5 US dollars) for their time and transportation. Women who had a Pap result of ASCUS or higher (n = 33, 2.1%) were referred to OB/GYN hospitals in HCMC for further examination and diagnosis. The parent study protocol was approved by Ethical Review Committees of the University of Medicine and Pharmacy in HCMC and of the Department of Science and Technology in HCMC. Given the objectives of the parent study, a desired sample size of 1,566 was identified. A total of 1,566 eligible women were contacted, of whom 1,550 agreed to participate (response rate = 99%). Fifty participants who had no HPV test results were excluded from this analysis, resulting in a total sample size of 1,500.

### Measures

Socio-demographic and behavioral variables used in this analysis were obtained from self-reported data via brief face-to-face interviews. These were participants’ factors and their husbands’ factors. Participants’ factors included age, parity, education level, marital status, age of first sexual intercourse, history of sexually transmitted infections (STIs), smoking history, lifetime number of sexual partners, and overall regular condom use. The husbands’ factors included education level, smoking history, and lifetime number of sexual partners. Current vaginitis or cervicitis was obtained from participants’ gynecological examination records. Specimens for HPV testing were obtained by cervical swab, placed in a sterile tube, and transferred to the Biology-Cell Laboratory of the University of Medicine and Pharmacy in HCMC on a daily basis for HPV DNA testing. L1 consensus PCR (MY09/11) was used to screen for HPV DNA. This test procedure could identify 25 HPV genotypes, including 8 low-risk types (6, 11, 42, 43, 61, 70, 71, 81), 16 high-risk types (16, 18, 31, 33, 35, 39, 45, 51, 52, 53, 56, 58, 59, 66, 68, 82), and one type with risk undetermined [17].

### Statistical analysis

To account for the complex survey design, the data used in this analysis were weighted for the population sizes of the primary sampling units (i.e., communes), aided by survey procedures in Stata 11 (StataCorp LP, College Station, Texas, USA). Frequency distributions were used to examine the prevalence of HPV infection with any types, high-risk types, and multiple types. Chi-square and binary logistic regression were used to examine associations between demographic/behavioral characteristics and high-risk-type HPV infections. The bivariate association between each demographic/behavioral characteristic and multi-type HPV infection was examined by an ordinal logistic regression [18], in which the dependent variable (HPV infection) contained three ordinal values: no infection, single-type infection, and multi-type (≥2 types) infection. Variables which altered the OR point estimates by ≥10% in bivariate analyses were included in the multivariable logistic regression models for adjusted associations with HPV infection. Univariable and multivariable associations with HPV infection were assessed using the adjusted F test. All variables used for this analysis had less than 3% of missing values; these were ignored in analyses.

## Results

Of the 1,500 surveyed respondents who had HPV test results, the mean age of participants was 42.4 years (SD = 9.5 years). About half of participants had two children and the highest number of children was nine (Table 1). The percentage of participants with education levels of secondary school or less was higher than that of their spouses (53.2% and 42.5%, respectively). The majority of participants were married (95.2%). Age of first intercourse ranged from 13 to 46 years (mean = 23.8, SD = 4.4). Four percent of participants had more than one sexual partner during their lifetime; 9% reported that their husbands had more than one sexual partner during their lifetime, and 18% did not know their husbands’ lifetime number of sexual partners. More than half of participants’ husbands ever smoked while only 1.3% of participants were smokers. In this sample, 88% of women reported that they did not use condoms or used condoms infrequently.

**Table 1:** **Prevalence and risk factors of high-risk HPV infections by demographic and behavioral characteristics**

**Characteristics**	**Total**		**High risk HPV infection**						
	**n** ^**a**^	**(%)** ^**b**^	**n** ^**a**^	**% high-risk HPV+**		**Unadjusted OR**		**Adjusted OR**	
				**(95% CI)** ^**b**^		**(95% CI)** ^**b,e**^		**(95% CI)** ^**b,e**^	
Total	1500		149	9.0	(5.4-12.6)				
Age	1500		149						
18-29	136	(9.1)	18	8.7	(3.5-14.0)	1		1	
30–39	454	(30.3)	35	4.6	(2.6-6.6)	.51	(.23-1.13)	.40	(.18-.90)
40–49	534	(35.6)	65	12.0	(4.6-19.4)	1.43	(.54-3.74)	.76	(.32-1.81)
≥50	376	(25.1)	31	10.1	(1.1-19.1)	1.17	(.35-3.86)	.74	(.27-2.01)
p value (for trend)							.056 (.181)		.101 (.545)
Parity	1500		149						
Nulliparous	54	(3.6)	4	5.3	(0.9-11.4)	.61	(.16-2.23)		
1	365	(24.3)	41	8.4	(5.1-11.7)	1			
2	669	(44.6)	53	9.6	(3.0-16.1)	1.15	(.48-2.76)		
≥3	412	(27.5)	51	8.9	(2.4-15.4)	1.07	(.43-2.64)		
p value (for trend)							.849 (.804)		
Education level of participant	1499		149						
Secondary school or lower	797	(53.2)	79	7.7	(3.1-12.3)	1			
High school or higher	702	(46.8)	70	11.3	(5.5-17.0)	1.52	(.64-3.62)		
p value							.340		
Education level of husband	1448		144						
Secondary school or lower	615	(42.5)	67	9.5	(3.4-15.5)	1			
High school or higher	833	(57.5)	77	8.4	(4.1-12.8)	.88	(.36-2.18)		
p value							.787		
Marital status	1426		142						
Married	1358	(95.2)	134	8.1	(4.8-11.5)	1			
Widowed/divorced/never married	68	(4.8)	8	8.5	(1.2-15.8)	1.06	(.37-2.98)		
p value							.918		
Age of first intercourse	1491		149						
<20	378	(25.4)	34	7.2	(.7-13.8)	1		1	
20-26	744	(49.9)	77	6.2	(4.5-8.0)	.85	(.31-2.35)	1.40	(.80-2.45)
>26	369	(24.8)	38	18.1	(5.7-30.5)	2.83	(.78-10.23)	4.69	(1.83-12.03)
p value (for trend)							**.030** (.159)		**.006 (.005)**
Participant ever smoked cigarettes	1493		149						
Yes	20	(1.3)	5	63.9	(21.2-106.7)	20.55	(3.07-137.76)	24.24	(3.26-180.24)
No	1473	(98.7)	144	7.9	(4.8-11.1)	1		1	
p value							**.002**		**.002**
Husband ever smoked cigarettes^c^	1493		149						
Yes	838	(56.1)	81	6.8	(3.2-10.4)	.50	(.21-1.20)	.68	(.31-1.49)
No	655	(43.9)	68	12.6	(5.3-19.9)	1		1	
p value							.123		.340
History of STIs ^d^	1179		118						
No	1091	(92.5)	109	8.4	(4.9-11.9)	1			
Yes	88	(7.5)	9	10.7	(4.5-25.8)	1.31	(.25-6.82)		
p value							.751		
Current vaginitis or cervicitis	1499		149						
No	1353	(90.3)	130	9.4	(5.3-13.5)	1			
Yes	146	(9.7)	19	6.1	(2.3-9.8)	.63	(.28-1.41)		
p value							.258		
Lifetime number of sexual partners	1486		146						
1	1423	(95.8)	140	9.1	(5.3-12.9)	1			
>1	63	(4.2)	6	4.3	(0.2-8.7)	.45	(.14-1.44)		
p value							.177		
Husband’s lifetime number of sexual partners	1494		147						
1	1092	(73.1)	107	8.1	(4.8-11.5)	1			
>1	135	(9.0)	10	7.9	(3.3-19.1)	.96	(.19-4.80)		
Don’t know	267	(17.9)	30	13.5	(.9-26.0)	1.76	(.55-5.64)		
p value							.633		
Overall condom use	1494		148						
Regular	178	(11.9)	8	3.6	(.5-6.7)	.36	(.13-.99)	.41	(.17-1.03)
Not regular/ Do not use	1316	(88.1)	140	9.4	(5.2-13.6)	1		1	
p value							**.049**		.057

^a^Number of unweighted participants.

^b^After weighting was applied.

^c^In participants who never smoked.

^d^Included Chlamydia, Gonorrhea, Syphilis, and Trichomoniasis.

^e^Odds ratio from logistic regressions.

**Figure 1:**
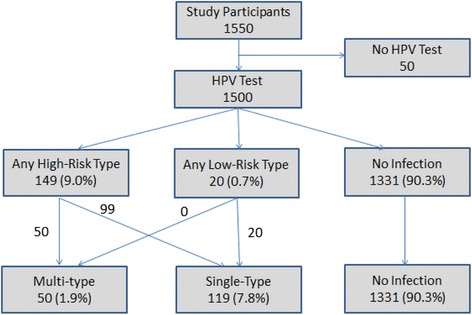
**HPV test results among participants.** Note: Numbers were based on unweighted counts and proportions were calculated after weighting.

The prevalence of HPV was 9.7% for any-type infection, 9.0% for high-risk infection, and 1.9% for multi-type HPV infections (Figure 1). Among those who were infected with any type of HPV, the proportions of high-risk HPV infection was 84% (due to a higher total number of high-risk types detected by this test), and of multi-type HPV infection was 20%. For multi-type HPV cases, all were high risk-type, the most common two-type HPV concurrence was 16 & 18 (25/43, 58.1%) and 16 & 58 (2/43, 4.6%). The 16 &18 pair was also present in 4/7 (57%) cases who were concurrently infected with three or more types of HPV. No case was infected with four types or more.

Table 1 presents bivariate associations between demographic and behavioral characteristics and HPV infections with any high-risk type(s). Participants’ age of first intercourse, lifetime smoking status, and frequency of condom use were significantly associated with high-risk HPV infection when compared to no infection. Because of the limited sample size of low risk type cases (n = 20), they were omitted from the analyses. Table 2 shows the prevalence of multi-type HPV infections by demographic and behavioral characteristics. Results from ordinal logistic regression in bivariate analyses suggested that HPV infections with one type (versus no infection) and with multi-type (versus with one type) were associated with participants’ smoking status and with irregular/no condom use. Prevalence of multi-type HPV infections varied across age groups of first intercourse (p = .030) but no specific trend could be confirmed (p-value for trend = .159). There was no difference in multi-type HPV infection by other characteristics of participants (e.g., age, parity, and lifetime number of sexual partners), and by characteristics of participants’ husbands (e.g., smoking status and lifetime number of sexual partners).

**Table 2:** **Prevalence and risk factors of multi-type HPV infections by demographic and behavioral characteristics**

**Characteristics**	**Multi-type HPV infection**						
	**n** ^**a**^	**% among HPV+**		**Unadjusted odd ratios**		**Adjusted odd ratios**	
		**(95% CI)** ^**b**^		**(95% CI)** ^**b,e**^		**(95% CI)** ^**b,e**^	
Total	50	20.1	(10.8-29.4)				
Age	50						
18-29	10	31.3	(10.5-52.1)	1		1	
30–39	18	37.1	(19.9-54.4)	0.52	(.24-1.13)	.43	(.20-.91)
40–49	15	14.3	(3.5-25.2)	1.34	(.54-3.53)	.74	(.34-1.63)
≥50	7	16.4	(.1-34.0)	1.12	(.37-3.41)	.74	(.30-1.84)
p value (for trend)					.061 (.184)		.122 (.578)
Parity	50						
Nulliparous	1	16.4	(4.1-46.9)	.59	(.18-1.98)		
1	16	33.6	(18.4-48.8)	1			
2	19	15.2	(3.1-27.3)	1.02	(.46-2.28)		
≥3	14	18.4	(2.3-34.5)	.89	(.37-2.13)		
p value (for trend)					.847 (.865)		
Education level of participant	50						
Secondary school or lower	27	19.1	(6.1-32.1)	1			
High school or higher	23	21.2	(8.2-34.3)	1.63	(.74-3.57)		
p value					.224		
Education level of husband	47						
Secondary school or lower	23	18.2	(4.1-32.4)	1			
High school or higher	24	19.9	(8.2-31.6)	1.01	(.44-2.34)		
p value					.981		
Marital status	49						
Married	45	22.0	(11.6-32.4)	1			
Widowed/divorced/never married	4	42.0	(10.3-73.6)	1.28	(.48-3.42)		
p value					.630		
Age of first intercourse	50						
<20	11	18.2	(5.8-37.6)	1		1	
20-26	29	30.8	(20.8-40.8)	.98	(.37-2.60)	1.62	(.96-2.74)
>26	10	11.9	(4.1-23.8)	2.85	(.86-9.48)	4.58	(1.94-10.83)
p value (for trend)					**.037** (.178)		**.002** (**.002**)
Participant ever smoked cigarettes	50						
Yes	1	22.8	(12.7-32.9)	12.52	(3.90-40.22)	15.36	(3.77-62.62)
No	49	2.0	(0.3-7.3)	1		1	
p value					**<.001**		**<.001**
Husband ever smoked cigarettes^c^	50						
Yes	27	18.2	(5.5-30.8)	.52	(.24-1.16)	.70	(.35-1.38)
No	23	22.3	(8.9-35.6)	1		1	
p value					.109		.302
History of STIs^d^	35						
No	29	17.1	(.6-44.9)	1			
Yes	6	18.3	(9.0-27.5)	1.20	(.25-5.70)		
p value					.823		
Current vaginitis or cervicitis	50						
No	46	20.0	(10.0-30.1)	1			
Yes	4	20.5	(18.0-39.1)	.65	(.30-1.39)		
p value					.265		
Lifetime number of sexual partners	49						
1	48	20.0	(12.1-31.1)	1			
>1	1	8.6	(1.1-43.8)	.67	(.23-1.93)		
p value					.288		
Husband’s lifetime number of sexual partners	49						
1	33	23.0	(11.5-34.5)	1			
>1	4	10.7	(.5-27.8)	1.01	(.24-4. 16)		
Don’t know	12	18.1	(.9-37.0)	1.83	(.65-5.13)		
p value					.515		
Overall condom use	49						
Regular	3	19.6	(10.0-29.1)	.35	(.13-.92)	.39	(.16-.92)
Not regular/ Do not use	46	26.3	(.3-55.9)	1		1	
p value					**.033**		**.032**

^a^Number of unweighted participants.

^b^After weighting was applied.

^c^In participants who never smoked.

^d^Included Chlamydia, Gonorrhea, Syphilis, and Trichomoniasis.

^e^Odds ratio from ordinal logistic regressions, in which we compare the odds of having multi-type HPV infection to the odds of having single-type HPV infection, and the odds of having single-type HPV infection to the odds of having no HPV infection.

Based on findings from previous studies and the 10% change-in-estimate rule, we included participants’ age, participants’ age of first intercourse, participants’ lifetime smoking, husbands’ lifetime smoking and condom use in multivariable models. The association between age of first intercourse and high-risk HPV infection and multi-type HPV infection remained significant (all p-values < .05). Participants’ smoking status was strongly and significantly associated with the two HPV-infection outcomes. Using no HPV infection as the reference group, the odds of being infected with high-risk-type HPV were 24.2 in women who ever smoked compared to those who never smoked. Smoking also increased the odds of being infected with multiple types of HPV (OR = 15.4). Regular condom use was significantly associated with a lower likelihood of multi-type HPV infection (p = .032). When we further compared 50 multi-type high risk cases with 99 single-type high risk HPV cases, the results were comparable to previous findings.

## Discussion

Our study results revealed that the prevalence of HPV in a population-based sample of women in HCMC, Vietnam was 9.0% for infection with high-risk types, and 1.9% for infection with multiple types of HPV. The prevalence of high-risk HPV infection in our study is comparable to previous results of vaginal-cervical HPV infection in a similar population in HCMC [5,7]; however, the prevalence of multi-type HPV infection is lower (1.9%) in our study compared to 4.5% in the IARC data in HCMC [5]. As reported in a previous publication using the same dataset, the most prevalent high-risk types of HPV were 16, 18, and 58 (which accounted for 40.7%, 26.2%, and 8.1% of all HPV infections, respectively) [16]. In this analysis, these three HPV types were also the most prevalent in multi-type HPV infection. There is no strong evidence to support type-clustering tendency in multiple infections [7,19]. In this study, the high prevalence of 16 &18 pairs was likely due to common behavioral risk factors for all HPV types, rather than because of the possibility of a biological interaction or clustering between 16 &18 types. Also, it was suggested that co-infecting HPV types might be related to HPV genetic similarity and to the testing procedure, which might be more likely to detect or miss certain HPV types with similar gene(s). For example, enzyme immunoassay, which was used in our study, is more likely to detect the excess of multiple HPV infections, compared to reverse line blot analysis [7].

Intervention or management to reduce high-risk HPV infection is important because of its oncogenic potential. Several studies have examined risk factors of high-risk HPV infection at the vaginal or cervical sites; however, evidence regarding risk factors other than sexual behavioral risks is inconsistent [6]. Although low education, high parity, and a history of STIs (e.g., Chlamydia or HSV-2) have been found to be associated with high-risk HPV infection [6,20-22], these associations were not observed in our study. Age of first sexual intercourse, which was also inconsistently related to HPV risk in previous studies, was associated with HPV prevalence in our results. In multivariable logistic regression models, our results suggested that older age of first sexual intercourse was a risk factor for high-risk HPV infection and multiple HPV infection. The effect of condom use in preventing HPV transmission and infection has not been fully established, although more studies have suggested a protective effect [22]. Our findings showed that regular condom use was associated with a lower likelihood of both high-risk-type and multi-type HPV infections.

Similar to several previous studies [23,24], our findings indicated that smoking was strongly associated with increased risk of HPV infection - with high-risk types or multiple types. Most Vietnamese women generally do not smoke; the prevalence of current tobacco smokers in reproductive-aged women was 0.8% [25]. Targeting smoking is particularly important because smoking does not only increase the likelihood of HPV infection but also contributes to the progression to HPV-related cancers [23,24]. Therefore, interventions to reduce smoking in women are needed for cervical cancer prevention. In this analysis, however, we could not examine tobacco use in detail (e.g., current smoking, numbers of cigarette smoked per day) due to the limited type of smoking related variables in the data set. Further studies are needed to explore the role of tobacco use and HPV infection in this population.

Although the proportions of multi-type HPV infection were not statistically different by most demographic and behavioral characteristics (possibly due to the small number of observed cases), the significant odds ratios of ordinal logistic regression suggested that irregular condom use and smoking increased the risk of being infected with multiple HPV types. In other words, risk factors of multi-type HPV infection in this study were very similar to the risk factors of high-risk type HPV infections. Although previous studies suggested that multi-type HPV infection was particularly more common in young women and women with multiple sexual partners [7,8,26], these associations were not observed in our results. The non-association between lifetime numbers of sexual partners and multi-type HPV infection might be due to the small number of participants who had more than one sexual partner in our predominantly married-women sample, given the taboos on premarital sex and extramarital sex for women in Vietnam [27,28]. It may be interesting to examine risk factors of multi-type HPV infection in younger female populations, in which sexual attitudes and practices may have taken a more liberal turn [27,29]. It is also important for future research or intervention projects to conduct HPV testing with main male sexual partner(s) in order to more comprehensively investigate the risk factors for multi-type HPV infections.

Our study has some limitations. Given the cross-sectional nature of the data, causality between risk factors and HPV outcomes cannot be established. However, most genital HPV infection is current and transient [6]; so HPV infection is unlikely to precede lifetime behavioral risks. Moreover, as we discussed above, factors such as smoking or irregular condom use have been suggested as risk determinants of high-risk-type or multi-type HPV infections in other prospective studies. Sexual and other behavioral risk factors were obtained from self-reports and hence might be subject to recall bias or under-reporting. To reduce potential biases of self-report, interviews were conducted in a private place, and interviewers were trained to aid participants in recalling their behaviors in different ways. The reliability of self-report might have been more compromised for responses related to risk behaviors of participants’ male sexual partners. This might explain why some husbands’ risk factors, such as husbands’ lifetime numbers of sexual partners, were not associated with high-risk or multi-type HPV infections in our analysis, although they have been significantly associated with HPV infection in other studies [15]. We did neither screen participants for their recent treatments of HPV (e.g., condylome), which might have influenced their current HPV presence, nor exclude them. However, the prevalence of cases with symptomatic HPV infection (e.g., types 6 & 11) who might have recently received a treatment was very low in the general population; and treatments for asymptomatic HPV infection (i.e., most high-risk types) were not available or recommended in Vietnam.

Because in our study multi-type HPV infection included only high-risk types, future studies should investigate multi-type HPV infection which comprises of multiple high-risk only, multiple low-risk only, and multiple with both high-and low-risk. Despite our relatively large sample size, the low HPV prevalence, particularly with multiple types, limited statistical power to detect associations and hence generated wide confidence intervals. Future prospective cohort studies are also needed to longitudinally investigate risk factors of multi-type HPV infection, interactions, and persistence.

## Conclusions

Our population-based survey suggested that risk factors for multi-type HPV infection in women in HCMC, Vietnam, were similar to risk factors for high-risk-type HPV infections. Risk factors, which showed a statistically strong association, included women’s smoking status, and older age of first sexual debut. Regular condom use was inversely associated with high-risk and multi-type HPV infection. Future research and intervention projects (e.g., related to HPV vaccination) are needed to reduce high-risk-type and multi-type HPV infections in order to prevent consequent HPV-related cancers.

## Abbreviations

HPV: Human papillomavirus
HCMC: Ho Chi Minh City
IARC: International Agency for Research on Cancer
STIs: Sexually transmitted infections
